# Development of a GeXP-multiplex PCR assay for the simultaneous detection and differentiation of six cattle viruses

**DOI:** 10.1371/journal.pone.0171287

**Published:** 2017-02-06

**Authors:** Qing Fan, Zhixun Xie, Zhiqin Xie, Xianwen Deng, Liji Xie, Li Huang, Sisi Luo, Jiaoling Huang, Yanfang Zhang, Tingting Zeng, Sheng Wang, Jiabo Liu, Yaoshan Pang

**Affiliations:** Guangxi Key Laboratory of Veterinary Biotechnology, Guangxi Veterinary Research Institute, Nanning, Guangxi Province, China; University of Malaya, MALAYSIA

## Abstract

Foot-and-mouth disease virus (FMDV), Bluetongue virus (BTV), Vesicular stomatitis Virus (VSV), Bovine viral diarrheal (BVDV), Bovine rotavirus (BRV), and Bovine herpesvirus 1 (IBRV) are common cattle infectious viruses that cause a great economic loss every year in many parts of the world. A rapid and high-throughput GenomeLab Gene Expression Profiler (GeXP) analyzer-based multiplex PCR assay was developed for the simultaneous detection and differentiation of these six cattle viruses. Six pairs of chimeric primers consisting of both the gene-specific primer and a universal primer were designed and used for amplification. Then capillary electrophoresis was used to separate the fluorescent labeled PCR products according to the amplicons size. The specificity of GeXP-multiplex PCR assay was examined with samples of the single template and mixed template of six viruses. The sensitivity was evaluated using the GeXP-multiplex PCR assay on serial 10-fold dilutions of ssRNAs obtained via in vitro transcription. To further evaluate the reliability, 305 clinical samples were tested by the GeXP-multiplex PCR assay. The results showed that the corresponding virus specific fragments of genes were amplified. The detection limit of the GeXP-multiplex PCR assay was 100 copies/μL in a mixed sample of ssRNAs containing target genes of six different cattle viruses, whereas the detection limit for the Gexp-mono PCR assay for a single target gene was 10 copies/μL. In detection of viruses in 305 clinical samples, the results of GeXP were consistent with simplex real-time PCR. Analysis of positive samples by sequencing demonstrated that the GeXP-multiplex PCR assay had no false positive samples of nonspecific amplification. In conclusion, this GeXP-multiplex PCR assay is a high throughput, specific, sensitive, rapid and simple method for the detection and differentiation of six cattle viruses. It is an effective tool that can be applied for the rapid differential diagnosis of clinical samples and for epidemiological investigation.

## Introduction

Foot-and-mouth disease virus (FMDV), Bluetongue virus (BTV), Vesicular stomatitis Virus (VSV), Bovine viral diarrheal virus (BVDV), Bovine rotavirus (BRV), and Bovine herpesvirus 1 (IBRV) are common cattle infectious viruses [1,2]. It was reported recently that these infectious diseases increased the beef cattle mortality to 5% and lead to economic losses estimated at $200 billion during 2015 in China [3]. These viral diseases in cattle exhibit similar clinical symptoms and are difficult to differentiate from each other. FMDV, VSV, BTV, BVDV and IBRV infections display skin lesions of various degrees, including vesicular lesions, erythema, skin cracking, and necrosis on the mouth, feet, noses, cunnus and teats etc [4–10]. Moreover, FMDV, VSV, and BTV are listed on The World Organization for Animal Health (OIE) Terrestrial Animal Health Code and countries are obligated to report these diseases to OIE [11]. Bovine rotavirus infection in young cattle less than 6 months old may not show typical symptoms as of BVDV. Both BRV and BVDV are frequently exhibits acute watery diarrhea and emaciation [12,13]. Therefore, it is important to diagnose and differentiate these infectious viruses accurately for the rapid control and prevention strategies [14].

Currently, OIE recommends antigen capture ELISA, virus isolation, and PCR, including real-time PCR, for the laboratory diagnosis of these viruses (http://www.oie.int/). However, PCR with low sensitivity and real-time PCR with limited plexity. The GenomeLab Gene Expression Profiler (GeXP) analyzer is a multiplex gene expression analysis platform that integrates PCR with capillary electrophoresis separation based on the size of the amplified products, and was designed to allow for the high-throughput, robust and differential assessment of multiplexed expression profile of up to 30 genes in one tube [15–18]. GeXP multiplex PCR assay has been successfully used for the rapid identification and differentiation of several animal infectious diseases [19–23]. In this study, a GeXP analyzer-based multiplex PCR assay was developed for the specific detection of six cattle infectious viruses: FMDV, BTV, VSV, BVDV, BRV and IBRV so that the assay can be applied for rapid differential diagnosis of these viral agents from clinical samples to adopt preventive and control measures against the cattle infectious diseases.

## Materials and methods

### Ethical statement

This study was approved by the Institutional Animal Care and Use Committee (IACUC) of Guangxi Veterinary Research Institution (GVRI). Sample collections were conducted based on the protocol #2012C101 issued by IACUC of GVRI. Farm owners agreed the consent regarding to sample collection in a written format. Well-trained veterinarians collected samples from calves in approval farms.

### Pathogens and DNA/RNA extraction

The pathogens used in this study were listed in Table 1. The genomic DNA of bacteria and mycoplasma strains were extracted from culture by using MiniBEST Universal Genomic DNA Extraction Kit (TaKaRa, Dalian, China) according to the manufacturer’s protocol. Each virus’s genomic RNA was extracted from 200 μL of virus suspension or clinical samples using MiniBEST Universal RNA Extraction Kit (TaKaRa, Dalian, China) according to the manual. The extracted DNA/RNA were eluted in 30 μL of distilled water. The RNA was synthesized to cDNA via reverse transcription using the PrimerScriptTM cDNA Synthesis Kit (TaKaRa, Dalian, China) with random primers (Nona-deoxyribonucleotide mixture) according to the manual, then quantified at 260 nm using a Nano Drop 2000 (Thermo Fisher Scientific, Waltham, USA). All the DNA/RNA were stored at -70°C until used.

**Table 1:** Pathogens used and GeXP assay results.

Pathogen	Source	GeXP Results					
		FMDV	BTV	VSV	BVDV	BRV	IBRV
FMDV							
FMDV serotype A inactivated virus	YNCIQ	+	-	-	-	-	-
FMDV serotype O inactivated virus	YNCIQ	+	-	-	-	-	-
FMDV serotype AsiaIinactivated virus	YNCIQ	+	-	-	-	-	-
FMDV serotype A inactivated vaccine	LVRI	+	-	-	-	-	-
FMDV serotype O inactivated vaccine	LVRI	+	-	-	-	-	-
FMDV serotype AsiaI inactivated vaccine	LVRI	+	-	-	-	-	-
BTV							
BTV serotype 4 inactivated virus	YNCIQ	-	+	-	-	-	-
BTV serotype8 inactivated virus	YNCIQ	-	+	-	-	-	-
BTV serotype 9 inactivated virus	YNCIQ	-	+	-	-	-	-
BTV serotype 15 inactivated virus	YNCIQ	-	+	-	-	-	-
BTV serotype 17 inactivated virus	YNCIQ	-	+	-	-	-	-
BTV serotype 18 inactivated virus	YNCIQ	-	+	-	-	-	-
VSV							
VSV serotype New Jersey inactivated virus	YNCIQ	-	-	+	-	-	-
VSV serotype Indiana inactivated virus	YNCIQ	-	-	+	-	-	-
BVDV							
Oregon CV24 (BVDV-1)	CVCC	-	-	-	+	-	-
NADL (BVDV-1)	CVCC	-	-	-	+	-	-
AV68 (BVDV-1)	CVCC	-	-	-	+	-	-
GX-BVDV1 (BVDV-1)	GVRI	-	-	-	+	-	-
GX-BVDV2 (BVDV-1)	GVRI	-	-	-	+	-	-
GX-BVDV3 (BVDV-1)	GVRI	-	-	-	+	-	-
GX-BVDV4 (BVDV-1)	GVRI	-	-	-	+	-	-
GX-BVDV5 (BVDV-1)	GVRI	-	-	-	+	-	-
GX-BVDV6 (BVDV-1)	GVRI	-	-	-	+	-	-
GX-BVDV7 (BVDV-1)	GVRI	-	-	-	+	-	-
GX-BVDV8 (BVDV-1)	GVRI	-	-	-	+	-	-
GX-BVDV9 (BVDV-1)	GVRI	-	-	-	+	-	-
GX-BVDV10 (BVDV-1)	GVRI	-	-	-	+	-	-
GX-BVDV11 (BVDV-1)	GVRI	-	-	-	+	-	-
GX-BVDV12(BVDV-1)	GVRI	-	-	-	+	-	-
GX-BVDV13 (BVDV-1)	GVRI	-	-	-	+	-	-
GX-041 (BVDV-2)	GVRI	-	-	-	+	-	-
BRV							
NCDV	CVCC	-	-	-	-	+	-
BRV014	CVCC	-	-	-	-	+	-
GX-BRV1	GVRI	-	-	-	-	+	-
GX-BRV2	GVRI	-	-	-	-	+	-
GX-BRV3	GVRI	-	-	-	-	+	-
GX-BRV4	GVRI	-	-	-	-	+	-
GX-BRV5	GVRI	-	-	-	-	+	-
GX-BRV6	GVRI	-	-	-	-	+	-
GX-BRV7	GVRI	-	-	-	-	+	-
GX-BRV8	GVRI	-	-	-	-	+	-
IBRV							
AV20/Barta Nu/67	CVCC	-	-	-	-	-	+
AV21/BK125	CVCC	-	-	-	-	-	+
Reference strain							
PPRV inactivated virus	YNCIQ	-	-	-	-	-	-
ETEC							
GX-ETEC1	GVRI	-	-	-	-	-	-
GX-ETEC2	GVRI	-	-	-	-	-	-
GX-ETEC3	GVRI	-	-	-	-	-	-
Escherichia coli							
C83919/1676	CVCC	-	-	-	-	-	-
C83924/x114/83	CVCC	-	-	-	-	-	-
C83922/b41	CVCC	-	-	-	-	-	-
Mycoplasma bovis							
GX/MB1	GVRI	-	-	-	-	-	-
GX/MB2	GVRI	-	-	-	-	-	-
Mycobacterium bovis							
GXmt304	GVRI	-	-	-	-	-	-
GXmt397	GVRI	-	-	-	-	-	-
C680001	CVCC	-	-	-	-	-	-
Salmonellosis/GXsal71	GVRI	-	-	-	-	-	-

GVRI = Guangxi Veterinary Research Institute;

YNCIQ = Yunnan Entry-Exit Inspection and Quarantine Bureau;

LVRI = Lanzhou Veterinary Research Institute;

CVCC = Chinese Veterinary Culture Collection Center.

### Primers design

The GeXP-multiplex PCR assay included six pairs of chimeric primers, and each of chimeric primers consisted of a gene-specific primer for each virus’s conserved sequence fused at 5’ end to a universal primer. The conserved nucleotide sequences of six cattle infectious disease viruses from GenBank were aligned using MegAlign 7.0 software (DNAStar, USA). Gene-specific primers were designed using the “Primer premier 5.0”(PRMIER Biosoft international, Canada) according to the restrict design rules of GeXP-multiplex PCR primer. A BLAST search program of GenBank website was performed to verify oligonucleotide specificity. All primers were synthesized and HPLC purified by the Invitrogen Inc (Guangzhou, China). The details of the oligonucleotides for primers were listed in Table 2.

**Table 2:** Primer information.

Primer	Forward primer sequence(5’-3’)	Reverse primer sequence(5’-3’)	Amplicon size (bp)	Target region	Primer concentration (μmol/L)
BTV	AGGTGACACTATAGAATAAGGGTAACTCACAGCAAACTCAA	GTACGACTCACTATAGGGAGAGCAGCCTGTCCATCCC	136	VP7	0.2
FMDV	AGGTGACACTATAGAATAGCCGTGGGACCATACAGG	GTACGACTCACTATAGGGAAAGTGATCTGTAGCTTGGAATCTC	166	3D	0.2
IBRV	AGGTGACACTATAGAATAGCGTCATTTACAAGGAGAACATC	GTACGACTCACTATAGGGAATCTCGCCCATGCCCAC	188	gB	0.2
BRV	AGGTGACACTATAGAATACAGTGGCTTCCATTAGAAGCAT	GTACGACTCACTATAGGGAGGTCACATCCTCTCACTA	211	VP6	0.2
VSV	AGGTGACACTATAGAATAAAACTACTGGACGGGCTTGA	GTACGACTCACTATAGGGATGAGATGCCCAAATGTTGC	278	N	0.2
BVDV	AGGTGACACTATAGAATAGTGAGTTCGTTGGATGGC	GTACGACTCACTATAGGGATATGTTTTGTATAAGAGTTCATTTG	308	5’-UTR	2

Universal tag sequences were underlined. Chimeric primers were synthesized using universal primers and gene-specific primers.

### GeXP-multiplex PCR assay

The reaction system was created using the GeXP Start-up Kit (Beckman Coulter, Brea, USA) in a total volume of 20 μL containing 4 μL of Genome LabTM GeXP Start Kit 5 × PCR Buffer (containing 0.25 μM concentration of each universal tag primer: Tag-F: 5’-AGGTGACACTATAGAATA-3’ and Tag-R: 5’-GTACGACTCACTATAGGGA-3’, the 5’ end of forward universal primer was labeled with Cy5 fluorophore), 4 μL of MgCl_2_ (25 μM), 2 μL of mixed primers (the concentration of each primer was listed in Table 2), 10 U JumpStart Taq DNA polymerase (Sigma-Aldrich, USA), and 1 μL of cDNA (0.5 pg~0.5 ng). Nuclease-free water was then added to the PCR reaction to achieve a final volume of 20 μL.

GeXP-multiplex PCR was performed using the thermal cycler (Thermo, Milford, USA). The optimized GeXP-multiplex PCR amplification condition as followed: 95°C for 3 minutes; 10 cycles of 95°C for 30 seconds, 55°C for 30 seconds and 72°C for30 seconds; then 10 cycles of 95°C for 30 seconds, 65°C for 30 seconds and 72°C for 30 seconds; and 20 cycles of 95°C for 30 seconds, 53°C for 30 seconds and 72°C for 30 seconds; held at 4°C for conservation.

PCR product separation and analysis were performed by capillary electrophoresis using GenomeLab GeXP Genetic Analysis System (Beckman Coulter, Brea, USA) following previously described [20]. The fluorescently labeled amplicons were separated into distinct peaks on a electropherogram via GeXP high-resolution capillary electrophoresis and then identified by their respective sizes. The peaks were initially analyzed by fragment analysis module of the GeXP system 10.2 software (Beckman Coulter, Brea, USA).

### Standards preparation

The specific genes of six cattle viruses were amplified by using the primers listed in Table 2. The specific PCR amplicons for each virus were cloned into the pEASY-T1 vector (Transgen Biotech, China) for sequencing. Sequence data were analyzed and blasted in GenBank. The six recombinant plasmids carrying the partial gene from each virus (VP7 gene of BTV, 3D gene of FMDV, gB gene of IBRV, VP6 gene of BRV, N gene of VSV, 5’-UTR of BVDV) were linearized with restriction enzyme *SpeI* (Takara, Dalian, China) and then in vitro transcribed into ssRNA using a T7 RiboMAX^™^ Express Large Scale RNA production system kit (Promega, Madison, WI, USA). The DNA template was removed by digestion with DNase following the transcription reaction, and then removed unincorporated nucleotides by chromatography. The concentration of transcribed ssRNAs were measured at 260 nm using a NanoDrop 2000 (ThermoFisher Scientific, Waltham, USA), and copy number of transcribed ssRNAs were calculate according to previously described [22,23]. Serial 10-fold dilutions, containing each transcribed ssRNA ranging from 10^8^ copies/μL to 1 copies/μL, were stored at -70°C until used.

### Specificity and sensitivity of GeXP-multiplex PCR assay

The GeXP-mono PCR assay and GeXP-multiplex PCR assay were used to evaluate its specificity. The GeXP-mono PCR assay was performed using a single template (cDNA extracted from each virus listed in Table 1) along with a primer mixture of six sets of chimeric primers to determine the size of the amplification products for each virus. GeXP-multiplex PCR assay was performed using a mixture template containing cDNA of six viruses and a primer mixture to evaluate its cross-amplification in GeXP-multiplex PCR system. The other references strains of bacterial or viruses commonly found in cattle (listed in Table 1) were tested by the GeXP-multiplex PCR to confirm its specificity.

The sensitivity of the GeXP-mono PCR assay for single target gene was examined by serial 10-fold dilutions of each transcribed ssRNA ranging from 10^8^ to 10^0^ copies/μL. The sensitivity of the GeXP-multiplex PCR assay was also examined by serial 10-fold dilutions of premixed templates, containing same copies of each transcribed ssRNA (FMDV, BTV, VSV, BVDV, BRV, and IBRV), which contained the specific gene sequences of the six cattle infectious viruses. The standards of the mixed template used ranging from 10^8^ to 10^0^ copies/μL were prepared from stock using serial 10-fold dilutions in RNase-free H_2_O and were used as templates to test the sensitivity of the new assay. One μL standard cDNA (0.5 pg~0.5 ng) was used in the reaction system. Profile of the reaction was described in GeXP-multiplex PCR assay section.

### Interference assay

The presence of other templates in high quantities could suppress the amplification of other low concentration templates and alter the amplification efficiency of GeXP-multiplex PCR. Two artificial samples containing various concentration of transcribed ssRNAs were prepared, mixed and detected by the GeXP-multiplex PCR assay to assess the interference between high concentration and low concentration nucleic acid templates. The results were compared with those of a single-template GeXP-multiplex PCR assay.

### Application to field samples

Three hundred and five field samples, including 156 fecal swabs, 30 conjunctival swabs, 30 nasal mucus swabs, 70 blood samples, 2 oesophageal-pharyngeal fluid, 2 vesicular fluid and tissue (10 mucous membranes, 2 vesicular skins, 3 lymph nodes) were collected from the various cattle farms in Guangxi, China during 2012 to 2014. More than three quarters of samples collected from cattle that did not have any typical clinical and pathological symptoms. A quarter of the samples were collected from diseased cattle showing different symptoms of diseases including metal lassitude, rhinorrhea, dysphagia, high fever, oral erosion, blisters and foaming at the mouth. The swab samples were placed into 1 mL sterilized water. Then supernatant was used to extract RNA after centrifugation. The liquid samples were used for the extraction of RNA as described previously. The tissue samples were ground into homogenates for RNA extraction. RNA was reverse-transcribed as described previously. The cDNA were assayed by both the optimized GeXP-multiplex PCR assay and simplex real-time PCR assays using previously published primers [24–29]. These simplex real-time PCR assays included five OIE recommended real-time PCR assays for detection of BTV, FMDV, IBRV, VSV, BVDV and one simplex real-time PCR for detection of BRV. All the positive field samples detected by the Gexp-multiplex PCR products were confirmed by DNA sequencing using conventional simplex PCR assays with same primers as the Gexp-multiplex PCR assay (Huada, Guangzhou, China).

## Results

### Specificity results

**Fig 1:**
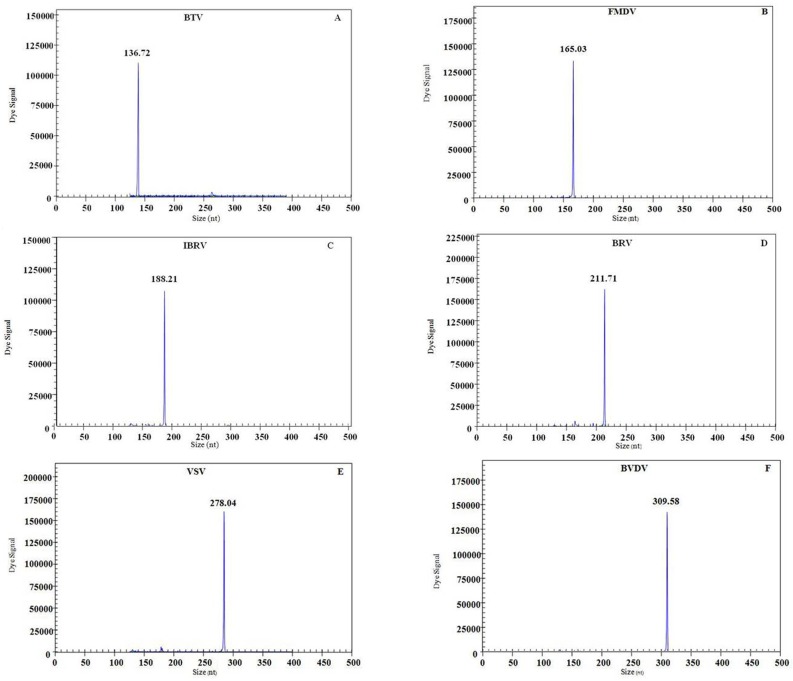
Specificity results of the GeXP-mono PCR assay. A-F showed the results of the amplifications of BTV, FMDV, IBRV, BRV, VSV, and BVDV, respectively. The Y-axis indicates the dye signal, and X-axis indicate the PCR product size.

**Fig 2:**
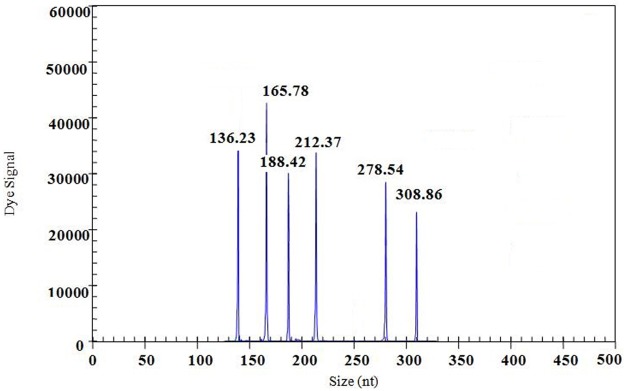
Specificity results of the GeXP-multiplex PCR assay with mixed template of six cattle infectious viruses.

The cDNA samples from six cattle infectious viruses listed in Table 1 were individually used as a template to evaluate the specificity of gene-specific primers. In GeXP-mono PCR assay, each of the corresponding genes from the target viruses was amplified as expected (Table 1 and Fig 1): BTV: 135~137 bp, FMDV: 165~167 bp, IBRV: 187~189 bp, BRV: 211~213 bp, VSV: 277~279 bp, BVDV: 308~310 bp. In GeXP-multiplex PCR assay, six specific amplification peaks generated by each target virus were detected simultaneously (Fig 2): BTV: 136.23 bp, FMDV: 165.78 bp, IBRV: 188.42 bp, BRV: 212.37 bp, VSV: 278.54 bp, BVDV: 308.86 bp. No cross amplification peak was observed in GeXP-mono PCR assay and GeXP-multiplex PCR assay. The GeXP-multiplex PCR assay specifically amplified six cattle infectious viruses, and exhibits no cross-reactivity with other cattle pathogens (Table 1). The results indicated that GeXP-multiplex PCR has a high specificity to detect six cattle infectious agents without any nonspecific amplification.

### Sensitivity results

**Fig 3:**
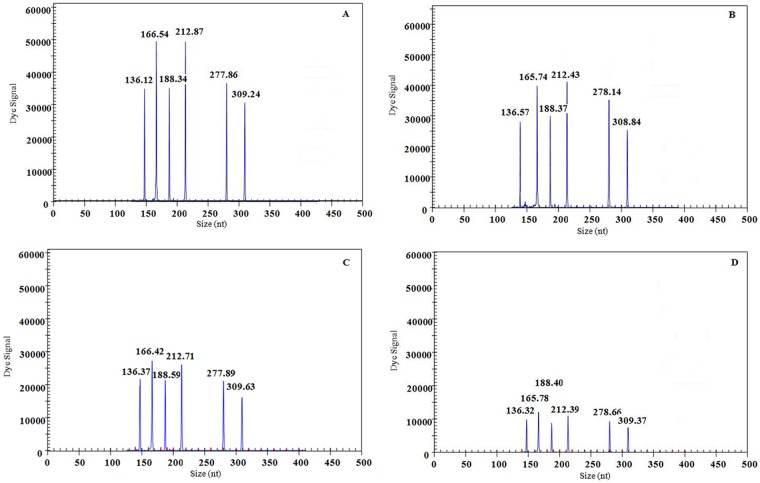
Sensitivity results of GeXP-multiplex PCR assay. GeXP-multiplex PCR assay was performed using serial 10-fold dilutions of premixed transcribed ssRNAs containing specific gene sequences of the 6 cattle viruses. A-D showed the results of equal amounts of template: 10^5^, 10^4^, 10^3^, 10^2^ copies per reactions in the GeXP-multiplex PCR assay. The viruses targets form left to right were as follow: BTV, FMDV, IBRV, BRV, VSV and BVDV.

The detection limit for the Gexp-mono PCR assay for a single target gene was 10 copies/μL of each transcribed ssRNA (data not shown). The sensitivity of the GeXP-multiplex PCR assay was examined using premixed ssRNAs mixtures with adjusted equal copies of each virus. The detection limit of the GeXP-multiplex PCR assay was 100 copies/μL when all of six premixed ssRNAs containing target genes of 6 cattle viruses were tested (Fig 3). Each tests were repeated three times at each template concentration and similar results were obtained. Typically the cut-off CT value for positive and negative results was determined as 2000 A.U. value (absorbance unite) by default. The results indicated that the GeXP-multiplex PCR assay has a good sensitivity to detect six cattle infectious viruses at the same time.

### Interference results

**Fig 4:**
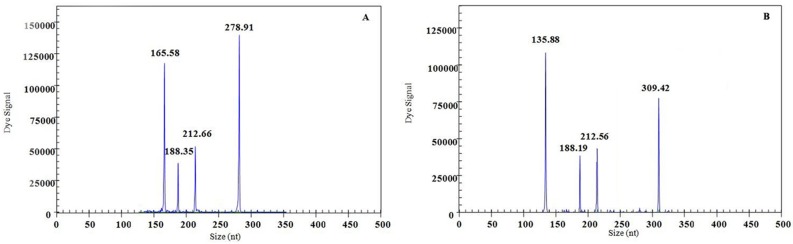
Interference results of GeXP-multiplex PCR assay. GeXP-multiplex PCR was carried out with the following artificial mixture samples: sample A: FMDV (10^6^ copies/μL) + IBRV (10^3^ copies/μL) + BRV (10^3^ copies/μL) + VSV (10^8^ copies/μL), sample B: BTV (10^7^ copies/μL) + IBRV (10^3^ copies/μL) + BRV (10^3^ copies/μL) + BVDV (10^5^copies/μL).

Two artificial samples: sample A:FMDV (10^6^ copies/μL) + IBRV (10^3^ copies/μL) + BRV (10^3^ copies/μL) + VSV (10^8^ copies/μL), sample B: BTV (10^7^ copies/μL) + IBRV (10^3^ copies/μL) + BRV (10^3^ copies/μL) + BVDV (10^5^ copies/μL), were prepared and tested by GeXP-multiplex PCR assay. The corresponding amplification peaks were observed in electrophoretogram (Fig 4). Additionally, the peaks of A.U. values of a simple template were similar to that of mixed templates (Table 3). Although there were some systematic deviations in the A.U. values, when comparing the mixed template with the single template, it did not affect the detection level. No differences in amplification efficiency were observed between the simple template and mixed template formats. The results suggest that variable viral concentration did not result in significant differences in amplification performance.

**Table 3:** Results of comparing the artificial mixed template with the single template by GeXP-multiplex PCR assay.

Template	A.U. value of GeXP-multiplex PCR assay					
	BTV	FMDV	IBRV	BRV	VSV	BVDV
Sample A		165.58	188.35	212.66	278.91	
FMDV (10^6^ copies/μL)		165.07				
IBRV (10^3^ copies/μL)			188.24			
BRV (10^3^ copies/μL)				212.15		
VSV (10^8^ copies/μL)					278.59	
Sample B	135.88		188.19	212.56		309.42
BTV (10^7^ copies/μL)	135.93					
IBRV (10^3^ copies/μL)			188.75			
BRV (10^3^ copies/μL)				212.21		
BVDV (10^5^ copies/μL)						309.57

### Detection in field samples

A total of 305 clinical samples were tested by the optimized GeXP-multiplex PCR assay and simplex real-time PCR assay to assess the reliability for the rapid detection of clinical samples. The positive and negative results obtained with the two different methods are shown in Table 4 and S1 Table. The detection rates for each virus were 10.5% (BTV), 2.0% (FMDV), 1.3% (IBRV), 2.6% (BRV), 0 (VSV), 13.4% (BVDV), respectively. The results of the GeXP-multiplex PCR assay has 100% agreement with simplex real-time PCR assays without any inconsistent results. Moreover, all positive samples in the GeXP-multiplex PCR and simplex real-time PCR were confirmed via sequencing to rule out false positive samples. This GeXP-multiplex PCR assay could detect and differentiate the six cattle viruses.

**Table 4:** Analysis of clinical samples using GeXP-multiplex PCR assay and simplex real-time PCR methods.

Background of clinical samples	Clinical sample	Number	Positive results (GeXP-multiplex PCR / simplex real-time PCR /sequencing)					
			BTV^a^	FMDV^a^	IBRV^a^	BRV^b^	VSV^a^	BVDV^a^
cattle without any morbid symptoms and signs	fecal swab	141				3/3/3		18/18/18
	blood sample	70	32/32/32					
	conjunctival swab	30						
	nasal mucus swab	22						
Cattle showed typical symptoms of disease	oesophageal-pharyngeal fluid	2		2/2/2				
	vesicular skins	2		2/2/2				
	vesicular fluid	2		2/2/2				
	mucous membrane	10						10/10/10
	Fecal sample	15				5/5/5/		10/10/10
	lymph node	3						3/3/3
	nasal mucus swab	8			4/4/4			

^a^ Confirmed by OIE recommended real-time PCR detection of BTV, FMDV, IBRV, VSV, BVDV[24–28].

^b^ Confirmed by simplex real-time PCR detection of BRV[29].

## Discussion

FMDV, BTV, VSV, BVDV, BRV, and IBRV are the six main cattle infectious viruses with a high infection rate and prevalent worldwide. Several global outbreaks have occurred in history, resulting in severe economic loss of stockbreeding and damage to international trade of animal products [14]. These diseases are potential threat to cattle industry. Therefore, a rapid, high-throughput and effective detection and differentiation technique is needed for the clinical diagnosis of these cattle viruses.

Although multiplex conventional PCR and multiplex fluorescence real-time quantitative PCR have been used for the detection of multiple viruses, they are limited by their high interference and fail to detect multiple target genes in one tube [30–32]. The GenomeLab Gene Expression Profiler (GeXP) analyzer is a novel multi-target, high-throughput detection technique that is capable of differentially assessing the expression profile of up to 30 genes in one tube based on analysis of amplicons size by capillary electrophoresis. The analytical procedure includes modified reverse transcription and PCR amplification, followed by capillary electrophoretic separation. Two-stage amplification using fluorescent dye-labeled universal tag primers reduces the interference among the primers, and inferior amplification and non-specific reaction. The GeXP-multiplex PCR assay has highly specificity and sensitivity. By far, the GeXP-multiplex PCR assay has been widely used in veterinary diagnostics and medical examination [19–23]. For example: simultaneous detection of sixteen human respiratory virus types/subtypes, 11 human papilloma viruses, nine serotypes of enteroviruses associated with hand, foot, and mouth disease, influenza A H1N1 virus has been reported [33–36]. Therefore, high-throughput detection and accurate identification of multiple viruses can be achieved by using this technique in large numbers of samples with limited amounts of starting material.

In this study, we have successfully established a GeXP-multiplex PCR assays that can simultaneously identify the FMDV, BTV, VSV, BVDV, BRV, and IBRV in a single reaction. The optimal detection limit of GeXP-multiplex PCR assay was 100 copies/μL when all of six premixed transcribed ssRNAs containing target genes of 6 bovine viruses. In detection of 305 clinical samples, the results of GeXP-multiplex PCR were consistent with that of simplex real-time PCR recommended by OIE. The subsequent analysis of positive samples by sequencing demonstrated that the GeXP-multiplex PCR assay had no false positive samples of non-specific amplification. Although two hundred sixty three samples were collected from cattle without any morbid symptoms and signs, 32 blood samples were positive for BTV, 3 fecal swabs were positive for BRV, and 18 fecal swabs were positive for BVDV by GeXP-multiplex PCR detection. This necessitates the epidemiological surveillance for BTV, BRV and BVDV in clinically normal cattle. Accurate diagnosis of BVDV positive cattle and timely elimination of them can be incorporated in the disease control of cattle herds programs to purify herd.

In practice, it only needs one single RNA extraction, one PCR, and one capillary electrophoresis, which will obtain detection results of six cattle viruses. Single capillary electrophoresis can analyze 96 samples at a time. This high-throughput advantage can meet the demand for a large scale of epidemiological investigation.

## Conclusion

The GeXP-multiplex PCR assay described in the present study will provide a high throughput diagnostic method with high specificity and sensitivity for the simultaneous identification of the six very important cattle viruses. GeXP-multiplex PCR assay may therefore be adopted for the molecular epidemiologic surveillance of cattle infectious diseases for designing effective disease-control programs.

## Supporting information

S1 Table Field samples detected by GeXP-multiplex PCR assay. (PDF)

## References

[1] Tomas JD, Simo FP. Rebhun’s Diseases of Dairy Cattle. 2st ed Elsevier (Singapore) Pte Ltd Press; 2009.

[2] Cernicchiaro N, White BJ, Renter DG, Babcock AH. Evaluation of economic and performance outcomes associated with the number of treatments after an initial diagnosis of bovine respiratory disease in commercial feeder cattle. Am J Vet Res. 2013; 74(2): 300–309. 10.2460/ajvr.74.2.300 23363358

[3] Wen W, Huang Z, Ye J. The current analysis of the status and prospect of cattle industry in China. China animal husbandry and veterinary abstract. 2016; 32(1): 45–46. (Chinese)

[4] Wernery U, Kinne J. Foot and mouth disease and similar virus infections in camelids: a review. Rev Sci Tech. 2012; 31(3): 907–918. 23520744 10.20506/rst.31.3.2160

[5] Sierra S, Davila M, Lowenstein P, Domingo E. Response of foot-and-mouth disease virus to increased mutagenesis: influence of viral load and fitness in loss of infectivity. J Virol. 2000; 74(18): 8316–8323. 10954530 10.1128/jvi.74.18.8316-8323.2000 PMC116341

[6] Brito BP, Rodriguez LL, Hammond JM, Pinto J, Perez AM. Review of the Global Distribution of Foot-and-Mouth Disease Virus from 2007 to 2014. Transbound Emerg Dis. 2015; Epub ahead of print. 10.1111/tbed.12373 25996568

[7] Maclachlan NJ. Bluetongue: history, global epidemiology, and pathogenesis. Prev Vet Med. 2011; 102(2): 107–111. 10.1016/j.prevetmed.2011.04.005 21570141

[8] Smith PF, Howerth EW, Carter D, Gray EW, Noblet R, Berghaus RD, et al Host predilection and transmissibility of vesicular stomatitis New Jersey virus strains in domestic cattle (Bos taurus) and swine (Sus scrofa). BMC Vet Res. 2012; 8: 183–191. 10.1186/1746-6148-8-183 23034141 PMC3514395

[9] Waldner CL, Kennedy RI. Associations between health and productivity in cow-calf beef herds and persistent infection with bovine viral diarrhea virus, antibodies against bovine viral diarrhea virus, or antibodies against infectious bovine rhinotracheitis virus in calves. Am J Vet Res. 2008; 69(7): 916–927. 10.2460/ajvr.69.7.916 18593246

[10] Santman-Berends IM, Mars MH, van Duijn L, van Schaik G. Evaluation of the epidemiological and economic consequences of control scenarios for bovine viral diarrhea virus in dairy herds. J Dairy Sci. 2015; 98(11):7699–7716. 10.3168/jds.2014-9255 26364098

[11] Word organization for animal health (OIE). The OIE List of Notifiable Terrestrial and Aquatic Animal Diseases 2016. http://www.oie.int/en/international-standard-setting/terrestrial-manual/access-online/.

[12] Xie JX, Duan ZJ, Li DD, Li BW, Lan B, Li YQ, et al Detection of bovine rotavirus G10P[11] in a diary farm in Daqing, China. Bing Du Xue Bao. 2010; 26(5): 407–409. (Chinese) 21043143

[13] Hashish EA, Zhang C, Ruan X, Knudsen DE, Chase CC, Isaacson RE, et al A multiepitope fusion antigen elicits neutralizing antibodies against enterotoxigenic Escherichia coli and homologous bovine viral diarrhea virus in vitro. Clin Vaccine Immunol. 2013; 20(7): 1076–1083. 10.1128/CVI.00249-13 23697572 PMC3697457

[14] Karreman HJ. Disease control on organic and natural cattle operations. Anim Health Res Rev. 2009; 10(2): 121–124. 10.1017/S1466252309990156 20003647

[15] Drew JE, Mayer CD, Farquharson AJ, Young P, Barrera LN. Custom design of a GeXP multiplexed assay used to assess expression profiles of inflammatory gene targets in normal colon, polyp, and tumor tissue. J Mol Diagn. 2011; 13(2): 233–242. 10.1016/j.jmoldx.2010.10.001 21354059 PMC3128578

[16] Yang MJ, Luo L, Nie K, Wang M, Zhang C, Li J, et al Genotyping of 11 human papillomaviruses by multiplex PCR with a GeXP analyzer. J Med Virol. 2012; 84(6): 957–963. 10.1002/jmv.23275 22499019

[17] Hu X, Zhang Y, Zhou X, Xu B, Yang M, Wang M, et al Simultaneously typing nine serotypes of enteroviruses associated with hand, foot, and mouth disease by a GeXP analyzer-based multiplex reverse transcription-PCR assay. J Clin Microbiol. 2012; 50(2): 288–293. 10.1128/JCM.05828-11 22116146 PMC3264198

[18] Rai AJ, Kamath RM, Gerald W, Fleisher M. Analytical validation of the GeXP analyzer and design of a workflow for cancer-biomarker discovery using multiplexed gene-expression profiling. Anal Bioanal Chem. 2009; 393(5): 1505–1511. 10.1007/s00216-008-2436-7 18958454

[19] Xie ZX, Luo SS, Xie LJ, Liu JB, Pang YS, Deng XW, et al Simultaneous typing of nine avian respiratory viruses using a novel GeXP analyzer-based multiplex PCR assay. J Virol Methods. 2014; 207:188–195. 10.1016/j.jviromet.2014.07.007 25025815

[20] Zhang YF, Xie ZX, Xie LJ, Deng XW, Xie ZQ, Luo SS, et al GeXP analyzer-based multiplex reverse-transcription PCR assay for simultaneous detection and differentiation of eleven duck viruses. BMC Microbiology. 2015; 15: 247–255. 10.1186/s12866-015-0590-6 26518004 PMC4628294

[21] Zhang MX, Xie ZX, Xie LJ, Deng XW, Xie ZQ, Luo SS, et al Simultaneous detection of six reproductive and respiratory swine viruses using a novel GeXP analyser-based multiplex PCR Assay. J Virol Methods. 2015; 224: 9–15. 10.1016/j.jviromet.2015.08.001 26259690

[22] Zeng TT, Xie ZX, Xie LJ, Deng XW, Xie ZQ, Luo SS, et al Simultaneous detection of six immunosuppressive chicken viruses by GeXP analyser-based multiplex PCR assays. Virol J. 2015; 12: 226–231. 10.1186/s12985-015-0455-5 26715327 PMC4696179

[23] Li M, Xie ZX, Xie ZQ, Liu JB, Xie LJ, Deng XW, et al Simultaneous detection of four different neuraminidase types of avian influenza A H5 viruses by multiplex reverse transcription-PCR using a GeXP analyse. Influenza Other Respir Viruses. 2016; 10(2): 141–149. 10.1111/irv.12370 26677838 PMC4746555

[24] Hofman M, Griot C, Chaignat V, Perler L, Thur B. Bluetongue disease reaches Switzerland. Schweiz Arch Tierheilk. 2008; 150: 49–56. 10.1024/0036-7281.150.2.49 18369049

[25] Shaw AE, Reid SM, Ebert K, Hutchings GH, Ferris NP, King DP. Protocol: Implementation of a one-step real-time RT-PCR protocol for diagnosis of foot-and-mouth disease. J Virol Methods. 2007; 143: 81–85. 10.1016/j.jviromet.2007.02.009 17397937

[26] Wilson WC, Letchworkth GJ, Jimenez C, Herrero MV, Navarro R, Paz P, et al Field evaluation of a multiplex real-time reverse transcription polymerase chain reaction assay for detection of Vesicular stomatitis virus. J Vet Diagn Invest. 2009; 21: 179–186. 19286495 10.1177/104063870902100201

[27] Hoffmann B, Depner K, Schirrmeier H, Beer M. A universal heterologous internal control system for duplex real-time RT-PCR assays used in a detection system for pestiviruses. J Virol Methods. 2006; 136: 200–209. 10.1016/j.jviromet.2006.05.020 16806503

[28] Wang J, O’Keefe J, Orr D, Loth L, Banks M, Wakeley P, et al An international inter-laboratory ring trial to evaluate a real-time PCR assay for the detection of bovine herpesvirus 1 in extended bovine semen. Vet Microbiol. 2008; 126: 11–19. 10.1016/j.vetmic.2007.06.005 17656045

[29] Otto PH, Rosenhain S, Elschner MC, Hotzel H, Machnowska P, Trojnar E, et al Detection of rotavirus species A, B and C in domestic mammalian animals with diarrhoea and genotyping of bovine species A rotavirus strains. Vet Microbiol. 2015; 179(30): 168–176. 26223422 10.1016/j.vetmic.2015.07.021

[30] Zeng Z, Liu Z, Wang W, Tang D, Liang H, Liu Z. Establishment and application of a multiplex PCR for rapid and simultaneous detection of six viruses in swine. J Virol Methods. 2014; 208: 102–106. 10.1016/j.jviromet.2014.08.001 25116201

[31] Park JY, Moon JS, Park SY, Song CS, Yeh JY, Lee JH, et al Simultaneous detection of rift valley fever, bluetongue, rinderpest, and peste des petits ruminants viruses by a single-tube multiplex reverse transcriptase-pcr assay using a dual-priming oligonucleotide system. Journal of Clinical Microbiology. 2011; 49(4): 1389–1394. 10.1128/JCM.00710-10 21307219 PMC3122808

[32] Fernández J, Agüero M, Romero L, Sánchez C, Belák S, Arias M, et al Rapid and differential diagnosis of foot-and-mouth disease, swine vesicular disease, and vesicular stomatitis by a new multiplex RT-PCR assay. J Virol Methods. 2008; 147(2): 301–311. 10.1016/j.jviromet.2007.09.010 17964668

[33] Li J, Mao NY, Zhang C, Yang MJ, Wang M, Xu WB, et al The development of a GeXP-based multiplex reverse transcription-PCR assay for simultaneous detection of sixteen human respiratory virus types/subtypes. BMC Infect Dis. 2012; 12: 189–194. 10.1186/1471-2334-12-189 22891685 PMC3462154

[34] Yang MJ, Luo L, Nie K, Wang M, Zhang C, Li J, et al Genotyping of 11 human papillomaviruses by multiplex PCR with a GeXP analyzer. J Med Virol. 2012; 84(6): 957–963. 10.1002/jmv.23275 22499019

[35] Hu X, Zhang Y, Zhou X, Xu B, Yang M, Wang M, et al Simultaneously typing nine serotypes of enteroviruses associated with hand, foot, and mouth disease by a GeXP analyzer-based multiplex reverse transcription-PCR assay. J Clin Microbiol. 2012; 50(2): 288–293. 10.1128/JCM.05828-11 22116146 PMC3264198

[36] Qin M, Wang DY, Huang F, Nie K, Qu M, Miao W, et al Detection of pandemic influenza A H1N1 virus by multiplex reverse transcription-PCR with a GeXP analyzer. J Virol Methods. 2010; 168(1–2): 255–258. 10.1016/j.jviromet.2010.04.031 20452377

