# Correction: Development of a GeXP-multiplex PCR assay for the simultaneous detection and differentiation of six cattle viruses

**DOI:** 10.1371/journal.pone.0173829

**Published:** 2017-03-07

**Authors:** Qing Fan, Zhixun Xie, Zhiqin Xie, Xianwen Deng, Liji Xie, Li Huang, Sisi Luo, Jiaoling Huang, Yanfang Zhang, Tingting Zeng, Sheng Wang, Jiabo Liu, Yaoshan Pang

There is an error in the Funding section. The correct funding information is as follows: This work was supported by the Guangxi Key Technologies Research and Development Program (0992033–5), Guangxi Government Senior Scientist Foundation (2011B020), and Guangxi Natural Science Foundation (2016GXNSFCA380003). The funders had no role in study design, data collection and analysis, decision to publish, or preparation of the manuscript.
