# Supplementary material for: Development of a GeXP-multiplex PCR assay for the simultaneous detection and differentiation of six cattle viruses
Source: PLoS One. 2017 Feb 6;12(2):e0171287. doi: 10.1371/journal.pone.0171287 (PMC5293189; doi:10.1371/journal.pone.0171287)
Supplement: S1 Table — (PDF) [file pone.0171287.s001.pdf]

S1 Table. Filed samples detected by GeXP-multiplex PCR assay

Sample No.	Sample type	Source	Results		
			GeXP-multiplex PCR	Simplex real-time PCR	Sequencing
1	oesophageal-pharyngeal fluid	Yellow cow/Guangxi/FCG897/2013	FMDV+	FMDV+	FMDV+
2	oesophageal-pharyngeal fluid	Yellow cow/Guangxi/FCG/Chengnan/2014	FMDV+	FMDV+	FMDV+
3	vesicular skins	Yellow cow/Guangxi/FCG897/2013	FMDV+	FMDV+	FMDV+
4	vesicular skins	Yellow cow/Guangxi/FCG/ Chengnan/2014	FMDV+	FMDV+	FMDV+
5	vesicular fluid	Yellow cow/Guangxi/FCG897/2013	FMDV+	FMDV+	FMDV+
6	vesicular fluid	Yellow cow/Guangxi/FCG/ Chengnan/2014	FMDV+	FMDV+	FMDV+
7	blood sample	Holstein/Guangxi/LZ113/2013	BTV+	BTV+	BTV+
8	blood sample	Holstein/Guangxi/LZ1417/2013	BTV+	BTV+	BTV+
9	blood sample	Holstein/Guangxi/LZ154/2013	BTV+	BTV+	BTV+
10	blood sample	Holstein/Guangxi/LZ115/2013	BTV+	BTV+	BTV+
11	blood sample	Holstein/Guangxi/LZ116/2013	BTV+	BTV+	BTV+
12	blood sample	Holstein/Guangxi/LZ177/2013			
13	blood sample	Holstein/Guangxi/LZ126/2013			
14	blood sample	Holstein/Guangxi/LZ218/2013	BTV+	BTV+	BTV+
15	blood sample	Holstein/Guangxi/LZ133/2013			
16	blood sample	Holstein/Guangxi/LZ169/2013			
17	blood sample	Holstein/Guangxi/LZ261/2013	BTV+	BTV+	BTV+
18	blood sample	Holstein/Guangxi/LZ201/2013			
19	blood sample	Holstein/Guangxi/LZ262/2013	BTV+	BTV+	BTV+
20	blood sample	Holstein/Guangxi/LZ203/2013			
21	blood sample	Holstein/Guangxi/LZ206/2013			
22	blood sample	Holstein/Guangxi/LZ246/2013	BTV+	BTV+	BTV+
23	blood sample	Holstein/Guangxi/LZ259/2013	BTV+	BTV+	BTV+
24	blood sample	Holstein/Guangxi/LZ4213/2014			
25	blood sample	Holstein/Guangxi/LZ4219/2014			
26	blood sample	Holstein/Guangxi/LZ4376/2014			
27	blood sample	Holstein/Guangxi/LZ4377/2014			
28	blood sample	Holstein/Guangxi/LZ4378/2014			
29	blood sample	Holstein/Guangxi/LZ4691/2014			
30	blood sample	Holstein/Guangxi/LZ4700/2014			
31	blood sample	Holstein/Guangxi/LZ4701/2014			
32	blood sample	Holstein/Guangxi/LZ4703/2014			
33	blood sample	Holstein/Guangxi/LZ4752/2014	BTV+	BTV+	BTV+
34	blood sample	Holstein/Guangxi/LZ4762/2014	BTV+	BTV+	BTV+

35	blood sample	Holstein/Guangxi/LZ4763/2014	BTV+	BTV+	BTV+
36	blood sample	Holstein/Guangxi/LZ1785/2014			
37	blood sample	Holstein/Guangxi/LZ1786/2014			
38	blood sample	Holstein/Guangxi/LZ1787/2014			
39	blood sample	Holstein/Guangxi/LZ1789/2014			
40	blood sample	Holstein/Guangxi/LZ1791/2014			
41	blood sample	Holstein/Guangxi/LZ609/2014	BTV+	BTV+	BTV+
42	blood sample	Holstein/Guangxi/LZ4711/2014			
43	blood sample	Holstein/Guangxi/LZ4712/2014			
44	blood sample	Holstein/Guangxi/LZ4715/2014			
45	blood sample	Holstein/Guangxi/LZ4716/2014			
46	blood sample	Holstein/Guangxi/LZ4717/2014			
47	blood sample	Holstein/Guangxi/LZ4718/2014			
48	blood sample	Holstein/Guangxi/LZ36/2013	BTV+	BTV+	BTV+
49	blood sample	Holstein/Guangxi/LZ37/2013	BTV+	BTV+	BTV+
50	blood sample	Holstein/Guangxi/LZ38/2013	BTV+	BTV+	BTV+
51	blood sample	Holstein/Guangxi/LZ391/2013			
52	blood sample	Holstein/Guangxi/LZ400/2013			
53	blood sample	Holstein/Guangxi/LZ41/2013	BTV+	BTV+	BTV+
54	blood sample	Holstein/Guangxi/LZ42/2013	BTV+	BTV+	BTV+
55	blood sample	Holstein/Guangxi/LZ512/2013	BTV+	BTV+	BTV+
56	blood sample	Holstein/Guangxi/LZ518/2013	BTV+	BTV+	BTV+
57	blood sample	Holstein/Guangxi/LZ521/2013	BTV+	BTV+	BTV+
58	blood sample	Holstein/Guangxi/LZ576/2013	BTV+	BTV+	BTV+
59	blood sample	Holstein/Guangxi/LZ613/2013			
60	blood sample	Holstein/Guangxi/LZ618/2013			
61	blood sample	Holstein/Guangxi/LZ622/2013			
62	blood sample	Holstein/Guangxi/LZ623/2013			
63	blood sample	Holstein/Guangxi/LZ624/2013	BTV+	BTV+	BTV+
64	blood sample	Holstein/Guangxi/LZ770/2013	BTV+	BTV+	BTV+
65	blood sample	Juanshan/Guangxi/LZ1120/2013	BTV+	BTV+	BTV+
66	blood sample	Juanshan/Guangxi/LZ1151/2013	BTV+	BTV+	BTV+
67	blood sample	Juanshan/Guangxi/LZ1123/2013	BTV+	BTV+	BTV+
68	blood sample	Juanshan/Guangxi/LZ1147/2013			
69	blood sample	Juanshan/Guangxi/LZ1170/2013			
70	blood sample	Juanshan/Guangxi/LZ930/2013	BTV+	BTV+	BTV+
71	blood sample	Juanshan/Guangxi/LZ845/2013			
72	blood sample	Juanshan/Guangxi/LZ846/2013			
73	blood sample	Juanshan/Guangxi/LZ847/2013	BTV+	BTV+	BTV+
74	blood sample	Juanshan/Guangxi/LZ23/2013			
75	blood sample	Juanshan/Guangxi/LZ13i/2013	BTV+	BTV+	BTV+
76	blood sample	Juanshan/Guangxi/LZ27i/2013	BTV+	BTV+	BTV+
77	nasal mucus	Water baffalo/Guangxi/GL19A/2014			

	swab				
78	nasal mucus swab	Water baffalo/Guangxi/GL32/2014			
79	nasal mucus swab	Water baffalo/Guangxi/GL/Ming17/2014			
80	nasal mucus swab	Water baffalo/Guangxi/GL/Cheng16/2014	IBRV+	IBRV+	IBRV+
81	nasal mucus swab	Water baffalo/Guangxi/GL/Cheng3/2014	IBRV+	IBRV+	IBRV+
82	nasal mucus swab	Water baffalo/Guangxi/GL/Cheng4/2014	IBRV+	IBRV+	IBRV+
83	nasal mucus swab	Water baffalo/Guangxi/GL/Cheng5/2014	IBRV+	IBRV+	IBRV+
84	nasal mucus swab	Water baffalo/Guangxi/GL/Zhang312/2014			
85	nasal mucus swab	Water baffalo/Guangxi/GL/Zhang46/2014			
86	nasal mucus swab	Water baffalo/Guangxi/GL/Wangdong40/2014			
87	nasal mucus swab	Water baffalo/Guangxi/GL/Wangdong24/2014			
88	nasal mucus swab	Water baffalo/Guangxi/GL/Zhangfuzhong21/2014			
89	nasal mucus swab	Water baffalo/Guangxi/GL/Zhangfuzhong1/2014			
90	nasal mucus swab	Water baffalo/Guangxi/GL/Zhangfuzhong003/2014			
91	nasal mucus swab	Water baffalo/Guangxi/GL/Jinggui14/2014			
92	nasal mucus swab	Water baffalo/Guangxi/GL/jinggui15/2014			
93	nasal mucus swab	Water baffalo/Guangxi/GL/jinggui16/2014			
94	nasal mucus swab	Water baffalo/Guangxi/GL/jinggui17/2014			
95	nasal mucus swab	Water baffalo/Guangxi/GL/jinggui18/2014			
96	nasal mucus swab	Holstein/Guangxi/BH002/2013			
97	nasal mucus swab	Holstein/Guangxi/BH012/2013			
98	nasal mucus swab	Holstein/Guangxi/BH045/2013			
99	nasal mucus	Holstein/Guangxi/BH046/2013			

	swab				
100	nasal mucus swab	Holstein/Guangxi/BH047/2013			
101	nasal mucus swab	Holstein/Guangxi/BH048/2013			
102	nasal mucus swab	Holstein/Guangxi/BH107/2013			
103	nasal mucus swab	Holstein/Guangxi/BH322/2013			
104	nasal mucus swab	Holstein/Guangxi/BH175/2013			
105	nasal mucus swab	Holstein/Guangxi/BH69/2013			
106	nasal mucus swab	Holstein/Guangxi/BH774/2013			
107	conjunctival swabs	Yellow cow/Guangxi/FCG/Lifugui07/2013			
108	conjunctival swabs	Yellow cow/Guangxi/FCG/Lifugui12/2013			
109	conjunctival swabs	Yellow cow/Guangxi/FCG/Lifugui49/2013			
110	conjunctival swabs	Yellow cow/Guangxi/FCG/Ling13/2013			
111	conjunctival swabs	Yellow cow/Guangxi/FCG/Ling06/2013			
112	conjunctival swabs	Yellow cow/Guangxi/FCG/Ling03/2013			
113	conjunctival swabs	Yellow cow/Guangxi/FCG/Ling08/2013			
114	conjunctival swabs	Yellow cow/Guangxi/FCG/LA85/2013			
115	conjunctival swabs	Yellow cow/Guangxi/FCG/LA89/2013			
116	conjunctival swabs	Yellow cow/Guangxi/FCG/Qian08/2022			
117	conjunctival swabs	Yellow cow/Guangxi/FCG/Qian01/2023			
118	conjunctival swabs	Yellow cow/Guangxi/FCG/Qian116/2013			
119	conjunctival swabs	Yellow cow/Guangxi/FCG/KM/2013			
120	conjunctival swabs	Holstein/Guangxi/FCG001/2012			
121	conjunctival	Holstein/Guangxi/FCG002/2012			

	swabs				
122	conjunctival swabs	Holstein/Guangxi/FCG003/2012			
123	conjunctival swabs	Holstein/Guangxi/FCG004/2012			
124	conjunctival swabs	Holstein/Guangxi/FCG005/2012			
125	conjunctival swabs	Holstein/Guangxi/FCG006/2012			
126	conjunctival swabs	Holstein/Guangxi/FCG007/2012			
127	conjunctival swabs	Holstein/Guangxi/FCG008/2012			
128	conjunctival swabs	Holstein/Guangxi/FCG009/2012			
129	conjunctival swabs	Holstein/Guangxi/FCG010/2012			
130	conjunctival swabs	Holstein/Guangxi/FCG011/2012			
131	conjunctival swabs	Holstein/Guangxi/FCG012/2012			
132	conjunctival swabs	Holstein/Guangxi/FCG013/2012			
133	conjunctival swabs	Holstein/Guangxi/FCG014/2012			
134	conjunctival swabs	Holstein/Guangxi/FCG015/2012			
135	conjunctival swabs	Holstein/Guangxi/FCG016/2012			
136	conjunctival swabs	Holstein/Guangxi/FCG017/2012			
137	mucous membrane	Holstein/Guangxi/HX/GX4/2013	BVDV+	BVDV+	BVDV+
138	mucous membrane	Holstein/Guangxi/HX013/2013	BVDV+	BVDV+	BVDV+
139	mucous membrane	Holstein/Guangxi/HX78/2013	BVDV+	BVDV+	BVDV+
140	mucous membrane	Holstein/Guangxi/HX67/2013	BVDV+	BVDV+	BVDV+
141	mucous membrane	Holstein/Guangxi/HX041/2013	BVDV+	BVDV+	BVDV+
142	mucous membrane	Holstein/Guangxi/HX07/2013	BVDV+	BVDV+	BVDV+
143	mucous	Holstein/Guangxi/NN6136/2012	BVDV+	BVDV+	BVDV+

	membrane				
144	mucous membrane	Holstein/Guangxi/NN6547/2012	BVDV+	BVDV+	BVDV+
145	mucous membrane	Holstein/Guangxi/NN0091/2012	BVDV+	BVDV+	BVDV+
146	mucous membrane	Holstein/Guangxi/NN2785/2012	BVDV+	BVDV+	BVDV+
147	lymph node	Holstein/Guangxi/HX/GX4/2013	BVDV+	BVDV+	BVDV+
148	lymph node	Holstein/Guangxi/HX013/2013	BVDV+	BVDV+	BVDV+
149	lymph node	Holstein/Guangxi/HX041/2013	BVDV+	BVDV+	BVDV+
150	fecal swab	Holstein/Guangxi/HX/GX4/2013	BVDV+	BVDV+	BVDV+
151	fecal swab	Holstein/Guangxi/HX013/2013	BVDV+	BVDV+	BVDV+
152	fecal swab	Holstein/Guangxi/HX78/2013	BVDV+	BVDV+	BVDV+
153	fecal swab	Holstein/Guangxi/HX67/2013	BVDV+	BVDV+	BVDV+
154	fecal swab	Holstein/Guangxi/HX041/2013	BVDV+	BVDV+	BVDV+
155	fecal swab	Holstein/Guangxi/HX07/2013	BVDV+	BVDV+	BVDV+
156	fecal swab	Holstein/Guangxi/HX001/2013			
157	fecal swab	Holstein/Guangxi/HX002/2013			
158	fecal swab	Holstein/Guangxi/HX003/2013			
159	fecal swab	Holstein/Guangxi/HX004/2013			
160	fecal swab	Holstein/Guangxi/HX005/2013			
161	fecal swab	Holstein/Guangxi/HX006/2013	BVDV+	BVDV+	BVDV+
162	fecal swab	Holstein/Guangxi/HX007/2013	BVDV+	BVDV+	BVDV+
163	fecal swab	Holstein/Guangxi/HX008/2013			
164	fecal swab	Holstein/Guangxi/HX009/2013			
165	fecal swab	Holstein/Guangxi/HX010/2013			
166	fecal swab	Holstein/Guangxi/HX011/2013	BVDV+	BVDV+	BVDV+
167	fecal swab	Holstein/Guangxi/HX012/2013	BVDV+	BVDV+	BVDV+
168	fecal swab	Holstein/Guangxi/HX013/2013			
169	fecal swab	Holstein/Guangxi/HX014/2013			
170	fecal swab	Holstein/Guangxi/HX015/2013	BVDV+	BVDV+	BVDV+
171	fecal swab	Holstein/Guangxi/HX016/2013			
172	fecal swab	Holstein/Guangxi/HX017/2013			
173	fecal swab	Holstein/Guangxi/HX018/2013	BVDV+	BVDV+	BVDV+
174	fecal swab	Holstein/Guangxi/HX019/2013			
175	fecal swab	Holstein/Guangxi/HX020/2013			
176	fecal swab	Holstein/Guangxi/HX021/2013			
177	fecal swab	Holstein/Guangxi/HX022/2013			
178	fecal swab	Holstein/Guangxi/HX023/2013			
179	fecal swab	Holstein/Guangxi/HX024/2013	BVDV+	BVDV+	BVDV+
180	fecal swab	Holstein/Guangxi/HX025/2013			
181	fecal swab	Holstein/Guangxi/HX026/2013	BVDV+	BVDV+	BVDV+
182	fecal swab	Holstein/Guangxi/HX027/2013			

183	fecal swab	Holstein/Guangxi/HX028/2013			
184	fecal swab	Holstein/Guangxi/HX029/2013			
185	fecal swab	Holstein/Guangxi/HX030/2013			
186	fecal swab	Holstein/Guangxi/HX031/2013			
187	fecal swab	Holstein/Guangxi/HX032/2013			
188	fecal swab	Holstein/Guangxi/HX033/2013	BVDV+	BVDV+	BVDV+
189	fecal swab	Holstein/Guangxi/HX034/2013			
190	fecal swab	Holstein/Guangxi/HX035/2013			
191	fecal swab	Holstein/Guangxi/HX036/2013			
192	fecal swab	Holstein/Guangxi/HX037/2013	BVDV+	BVDV+	BVDV+
193	fecal swab	Holstein/Guangxi/HX038/2013			
194	fecal swab	Holstein/Guangxi/HX039/2013			
195	fecal swab	Holstein/Guangxi/HX040/2013	BVDV+	BVDV+	BVDV+
196	fecal swab	Holstein/Guangxi/HX041/2013			
197	fecal swab	Holstein/Guangxi/HX042/2013			
198	fecal swab	Holstein/Guangxi/HX043/2013			
199	fecal swab	Holstein/Guangxi/HX044/2013			
200	fecal swab	Holstein/Guangxi/NN6136/2012	BVDV+	BVDV+	BVDV+
201	fecal swab	Holstein/Guangxi/NN6547/2012	BVDV+	BVDV+	BVDV+
202	fecal swab	Holstein/Guangxi/NN0091/2012	BVDV+	BVDV+	BVDV+
203	fecal swab	Holstein/Guangxi/NN2785/2012	BVDV+	BVDV+	BVDV+
204	fecal swab	Holstein/Guangxi/NN2103/2012			
205	fecal swab	Holstein/Guangxi/NN2245/2012			
206	fecal swab	Holstein/Guangxi/NN2185/2012			
207	fecal swab	Holstein/Guangxi/NN2324/2012	BVDV+	BVDV+	BVDV+
208	fecal swab	Holstein/Guangxi/NN2325/2012			
209	fecal swab	Holstein/Guangxi/NN2350/2012			
210	fecal swab	Holstein/Guangxi/NN289/2012			
211	fecal swab	Holstein/Guangxi/NN204/2012			
212	fecal swab	Holstein/Guangxi/NN206/2012			
213	fecal swab	Holstein/Guangxi/NN205/2012			
214	fecal swab	Holstein/Guangxi/NN242/2012			
215	fecal swab	Holstein/Guangxi/NN300/2012			
216	fecal swab	Holstein/Guangxi/NN963/2012			
217	fecal swab	Holstein/Guangxi/NN962/2012			
218	fecal swab	Holstein/Guangxi/NN3010/2012			
219	fecal swab	Holstein/Guangxi/NN3155/2012	BVDV+	BVDV+	BVDV+
220	fecal swab	Holstein/Guangxi/NN3176/2012			
221	fecal swab	Holstein/Guangxi/NN2785/2012			
222	fecal swab	Holstein/Guangxi/NN3986/2012			
223	fecal swab	Holstein/Guangxi/NN001/2013			
224	fecal swab	Holstein/Guangxi/NN002/2013			
225	fecal swab	Holstein/Guangxi/NN003/2013			

226	fecal swab	Holstein/Guangxi/NN004/2013			
227	fecal swab	Holstein/Guangxi/NN005/2013	BVDV+	BVDV+	BVDV+
228	fecal swab	Holstein/Guangxi/NN006/2013			
229	fecal swab	Holstein/Guangxi/NN007/2013			
230	fecal swab	Holstein/Guangxi/NN008/2013			
231	fecal swab	Holstein/Guangxi/NN009/2013			
232	fecal swab	Holstein/Guangxi/NN010/2013	BVDV+	BVDV+	BVDV+
233	fecal swab	Holstein/Guangxi/NN011/2013			
234	fecal swab	Holstein/Guangxi/NN012/2013			
235	fecal swab	Holstein/Guangxi/NN013/2013			
236	fecal swab	Holstein/Guangxi/NN014/2013	BRV+	BRV+	BRV+
237	fecal swab	Holstein/Guangxi/NN015/2013			
238	fecal swab	Holstein/Guangxi/NN016/2013	BRV+	BRV+	BRV+
239	fecal swab	Holstein/Guangxi/NN017/2013			
240	fecal swab	Holstein/Guangxi/NN018/2013			
241	fecal swab	Holstein/Guangxi/NN019/2013			
242	fecal swab	Holstein/Guangxi/NN020/2013			
243	fecal swab	Holstein/Guangxi/NN022/2013			
244	fecal swab	Holstein/Guangxi/NN023/2013			
245	fecal swab	Holstein/Guangxi/NN024/2013			
246	fecal swab	Holstein/Guangxi/NN025/2013			
247	fecal swab	Holstein/Guangxi/NN026/2013			
248	fecal swab	Holstein/Guangxi/NN027/2013			
249	fecal swab	Holstein/Guangxi/NN028/2013			
250	fecal swab	Holstein/Guangxi/NN029/2013	BVDV+	BVDV+	BVDV+
251	fecal swab	Holstein/Guangxi/NN030/2013			
252	fecal swab	Holstein/Guangxi/NN031/2013			
253	fecal swab	Holstein/Guangxi/NN032/2013			
254	fecal swab	Holstein/Guangxi/NN033/2013			
255	fecal swab	Holstein/Guangxi/NN034/2013			
256	fecal swab	Holstein/Guangxi/NN035/2013			
257	fecal swab	Holstein/Guangxi/NN036/2013			
258	fecal swab	Holstein/Guangxi/NN037/2013			
259	fecal swab	Holstein/Guangxi/NN038/2013			
260	fecal swab	Holstein/Guangxi/NN039/2013	BVDV+	BVDV+	BVDV+
261	fecal swab	Holstein/Guangxi/NN040/2013			
262	fecal swab	Holstein/Guangxi/NN041/2013			
263	fecal swab	Holstein/Guangxi/NN042/2013			
264	fecal swab	Holstein/Guangxi/NN043/2013			
265	fecal swab	Holstein/Guangxi/NN044/2013			
266	fecal swab	Holstein/Guangxi/NN045/2013			
267	fecal swab	Holstein/Guangxi/NN046/2013			
268	fecal swab	Holstein/Guangxi/NN047/2013			

269	fecal swab	Holstein/Guangxi/NN048/2013			
270	fecal swab	Holstein/Guangxi/NN049/2013			
271	fecal swab	Holstein/Guangxi/NN050/2013	BVDV+	BVDV+	BVDV+
272	fecal swab	Holstein/Guangxi/NN051/2013			
273	fecal swab	Holstein/Guangxi/NN052/2013			
274	fecal swab	Holstein/Guangxi/NN053/2013			
275	fecal swab	Holstein/Guangxi/NN054/2013			
276	fecal swab	Holstein/Guangxi/NN055/2013			
277	fecal swab	Holstein/Guangxi/NN056/2013			
278	fecal swab	Holstein/Guangxi/NN057/2013			
279	fecal swab	Holstein/Guangxi/NN058/2013	BRV+	BRV+	BRV+
280	fecal swab	Holstein/Guangxi/NN059/2013			
281	fecal swab	Holstein/Guangxi/NN060/2013			
282	fecal swab	Holstein/Guangxi/NN061/2013			
283	fecal swab	Holstein/Guangxi/NN062/2013			
284	fecal swab	Holstein/Guangxi/NN063/2013			
285	fecal swab	Holstein/Guangxi/NN064/2013			
286	fecal swab	Holstein/Guangxi/NN065/2013			
287	fecal swab	Holstein/Guangxi/NN001/2014			
288	fecal swab	Holstein/Guangxi/NN002/2014			
289	fecal swab	Holstein/Guangxi/NN003/2014			
290	fecal swab	Holstein/Guangxi/NN004/2014	BRV+	BRV+	BRV+
291	fecal swab	Holstein/Guangxi/NN005/2014			
292	fecal swab	Holstein/Guangxi/NN006/2014			
293	fecal swab	Holstein/Guangxi/NN007/2014			
294	fecal swab	Holstein/Guangxi/NN008/2014			
295	fecal swab	Holstein/Guangxi/NN009/2014			
296	fecal swab	Holstein/Guangxi/NN001/2014			
297	fecal swab	Holstein/Guangxi/NN010/2014	BRV+	BRV+	BRV+
298	fecal swab	Holstein/Guangxi/NN011/2014			
299	fecal swab	Holstein/Guangxi/NN012/2014	BRV+	BRV+	BRV+
300	fecal swab	Holstein/Guangxi/NN013/2014			
301	fecal swab	Holstein/Guangxi/NN014/2014			
302	fecal swab	Holstein/Guangxi/NN015/2014			
303	fecal swab	Holstein/Guangxi/NN016/2014	BRV+	BRV+	BRV+
304	fecal swab	Holstein/Guangxi/NN017/2014			
305	fecal swab	Holstein/Guangxi/NN018/2014	BRV+	BRV+	BRV+

FCG: Fangchenggang Cattle Farm, Fangchenggang, Guangxi Province;

NN: JingGuang Diary Farm, Nangning, Guangxi Province;

LZ: Huangshi Cattle Farm, Liuzhou, Guangxi Province;

BH: Beihai Cattle Farm, Beihai, Guangxi Province;

GL: Yongfu Cattle Farm, Guiling, Guangxi Province;

HX: Hengxian Cattle Farm, Hengxian, Guangxi Province;

+: positive.
